# Effects of Short-Term Warming and Altered Precipitation on Soil Microbial Communities in Alpine Grassland of the Tibetan Plateau

**DOI:** 10.3389/fmicb.2016.01032

**Published:** 2016-06-30

**Authors:** Kaoping Zhang, Yu Shi, Xin Jing, Jin-Sheng He, Ruibo Sun, Yunfeng Yang, Ashley Shade, Haiyan Chu

**Affiliations:** ^1^State Key Laboratory of Soil and Sustainable Agriculture, Institute of Soil Science, Chinese Academy of ScienceNanjing, China; ^2^Department of Ecology, College of Urban and Environmental Sciences and Key Laboratory for Earth Surface Processes of the Ministry of Education, Peking UniversityBeijing, China; ^3^Key Laboratory of Adaptation and Evolution of Plateau Biota, Northwest Institute of Plateau Biology, Chinese Academy of SciencesXining, China; ^4^State Key Joint Laboratory of Environment Simulation and Pollution Control, School of Environment, Tsinghua UniversityBeijing, China; ^5^Department of Microbiology and Molecular Genetics, Michigan State UniversityEast Lansing, MI, USA

**Keywords:** climate change, alpine grassland, soil microbial community structure, soil moisture, pyrosequencing

## Abstract

Soil microbial communities are influenced by climate change drivers such as warming and altered precipitation. These changes create abiotic stresses, including desiccation and nutrient limitation, which act on microbes. However, our understanding of the responses of microbial communities to co-occurring climate change drivers is limited. We surveyed soil bacterial and fungal diversity and composition after a 1-year warming and altered precipitation manipulation in the Tibetan plateau alpine grassland. In isolation, warming and decreased precipitation treatments each had no significant effects on soil bacterial community structure; however, in combination of both treatments altered bacterial community structure (*p* = 0.03). The main effect of altered precipitation specifically impacted the relative abundances of Bacteroidetes and Gammaproteobacteria compared to the control, while the main effect of warming impacted the relative abundance of Betaproteobacteria. In contrast, the fungal community had no significant response to the treatments after 1-year. Using structural equation modeling (SEM), we found bacterial community composition was positively related to soil moisture. Our results indicate that short-term climate change could cause changes in soil bacterial community through taxonomic shifts. Our work provides new insights into immediate soil microbial responses to short-term stressors acting on an ecosystem that is particularly sensitive to global climate change.

## Introduction

Global climate changes, whether caused by natural processes or anthropogenic activities, influence belowground organisms (Singh et al., 2010; Bellard et al., 2012), which in turn influence carbon and nitrogen cycling in terrestrial ecosystems. Although soil microbes represent a large fraction of belowground biomass and can regulate terrestrial carbon transformations, it remains unclear how the diversity and composition of soil microbial communities respond to global climate change stressors. Because nearly 90% of the microbes are yet uncultivable (Lok, 2015), many terrestrial climate change studies have focused on the responses of aggregate microbial characters, such as soil respiration, microbial biomass, or enzyme activity (Luo et al., 2001; Zhang et al., 2005; Suseela et al., 2014) to global climate change stressors. For those studies that have used more precise methods to measure microbial community composition (Evans et al., 2014; Xiong et al., 2014; Rui et al., 2015), it is uncommon for these studies to examine the influence of more than one climate change stressor in combination.

However, climate changes, such as warming and increased variability in rainfall, are frequently associated with each other (IPCC, 2013). The interactive impacts of warming and altered precipitation are of particular interest, as it has been suggested that precipitation could modify the effects of warming on microbial community composition (Zhang N. et al., 2015). When drought condition occurs, warming presented negative effect on microbial population size (Sheik et al., 2011). Multifactor studies focusing on plant communities have demonstrated that the interaction of warming and decreased precipitation resulted in a decrease in above-ground net primary production (Luo et al., 2008; Hoeppner and Dukes, 2012). However, how the associated below-ground microbial communities respond to both warming and altered precipitation is a particular gap in our knowledge of microbial community composition changes in response to these stressors. Because observed microbial responses have been complex in their direction and magnitude, their responses were inconsistent over time (Contosta et al., 2015). For example, there were pronounced shifts in microbial community structure by either warming or altered precipitation within 5-years (Horz et al., 2004; Gray et al., 2011; Zhang et al., 2013). However, in another similar study, soil bacterial and archaeal community composition were unchanged over 5-year of rainfall manipulation (Cruz-Martínez et al., 2009). After 1.5-year simulated warming, Xue et al. (2016) found no significant change in soil bacterial community structure in the active layer of tundra soil. Additionally, a 9-year warming experiment suggested that the soil microbial community structure was stable and resistant (Weedon et al., 2012), and an 11-year field irrigation experiment also did not observe effects on microbial community composition (Williams, 2007). Results from experiments investigating a gradient of warming (Rinnan et al., 2007; DeAngelis et al., 2015) suggested that more than a decade of stressor was needed to observe significant changes in microbial community structure, which the authors suggest is reflective of the time required for the anticipated gradual changes in below-ground community composition. However, a much shorter warming study (15 months) in a particularly sensitive ecosystem, the Tibetan plateau, suggested that the bacterial community structure was sensitive (Xiong et al., 2014). Thus, there is no consistency as to what minimal timescale is required to detect responses in soil microbial communities, or whether and how microbial communities respond to climate change more generally. In particular, short-term experimental investigations of co-occurring climate change stressors could provide key insights into how the diversity and composition of soil microbial communities respond to climate change.

The Tibetan plateau is the highest (average elevation 4000 m above sea level) and largest plateau (2.5 million km^2^) on Earth. This region has faced a twice faster rate than the average global warming rate of 0.2°C per decade over the past 50-years (Chen et al., 2013), as well as both increasing (in the southern and northern regions) and decreasing (in the central region) precipitation (You et al., 2008). Thus, the ecosystem is very fragile and sensitive to climate change. Extensive evidence shows that climate change stressors have strong effects on soil microbial community composition and function in the Tibetan grassland (Yang et al., 2014; Zhang B. et al., 2015). However, the response of microbial communities to the combination of altered precipitation and warming has not been investigated directly. Thus, we characterized the effects of short-term warming, altered precipitation and their interactions on soil microbial communities in the Tibetan grassland. We aimed to address two questions: (1) how do the bacterial and fungal communities respond to short-term warming, altered precipitation and their interactions; and (2) what are the key factors controlling microbial community variability after this combination of climate change stressors?

## Methods and materials

### Experimental design and soil sampling

The experimental site is located at the Haibei Alpine Grassland Ecosystem Research Station, in the northeast of the Tibetan Plateau (37°30' N, 101°12' E, 3200 m above sea level). This site has a typical plateau continental climate, average annual temperature ranges from −0.81 to −1.82°C (maximum 17.3°C, minimum −23.6°C) and annual precipitation ranges from 350.6 to 501.3 mm (as measured in 2009–2012), with more than 80% of the annual precipitation occurring in the growing season from May to September (Wang et al., 2014). The dominant plants are *Kobresia humilis, Festuca ovina, Elymus nutans, Poa pratensis, Carex scabrirostris, Scripus distigmaticus, Gentiana strminea, Gentiana farreri, Lenotop odiumnanum, Blvsmus sinocompressus, Potentilla nicea*, and *Dasiphora fruticosa* (Luo et al., 2010). The typical herbivorous animals are sheep and yaks. The soil is classified as a Cambisol (IUSS Working Group WRB, 2007).

Thirty-six 2 × 2 m plots (6 treatments × 6 replicates) were established in July, 2011. We used a randomized complete block design with warming and altered precipitation as main treatment factors, which included control, decreased precipitation (DP; −50%), increased precipitation (IP; +50%), warming (W; +~2°C), warming with decreased precipitation (W × DP), and warming with increased precipitation (W × IP). Each treatment had six replicates. To achieve warming, two parallel infrared heaters (1000 × 22 mm) hung 1.5 m above each warmed plot. The height of the heaters was adjusted to evenly heat the soil surface up (Kimball, 2005). The temperature of topsoil was monitored by EM50 (decagon devices, USA), which automatically measured the temperature hourly and were regulated to maintain ~2°C average temperature difference between the warmed and control plots. Four transparent resin poly carbonate channels (accounting for ~50% percent of the plot area) were set above the infrared heaters to collect precipitation in the decreased precipitation plots. When raining, the collected precipitation flowed into white polyethylene plastic rainwater collection vessels beside the plots, and this rainwater was then added to the increased precipitation plots (Figure S1). The control plot had two dummy heaters (no warming) and four transparent channels to control for shade effects of the equipment deployment. Iron sheets were buried around each plot to prevent runoff.

Soil samples were collected on September15th–16th in 2012. In each plot, five representative points (four vertices and one center) of the topsoil (0–5 cm in depth) were collected by drill (5 cm in diameter). At each sampling, the drill was cleaned, washed with sterile water, and then air dried. Samples from the same plot were pooled into one composite sample, packed in polyethylene bags, immediately stored in a cooler with ice packs, and shipped to the laboratory. The composite samples were sieved (2 mm), and all of the visible roots, residues, and stones were removed. Subsamples were stored at 4°C for the measurement of soil biogeochemical properties, and at −20°C for soil DNA extraction. We additionally selected three replicates of each treatment to record plant species in July 2012. Because this ecosystem is a protected area, we collected plants only as often as necessary to assess their community diversity.

### Soil biogeochemical properties measurement

Soil pH was measured with a fresh soil to water ratio of 1:5 by pH probe (FE20-FiveEasy™ pH, Mettler Toledo, Germany). Soil moisture was determined gravimetrically at 105°C for 6 h. Nitrate (${\text{NO}}_{3}^{-}$−N), ammonium (${\text{NH}}_{4}^{+}$−N), dissolved organic carbon (DOC) were extracted by adding 3 g fresh soil to 30 mL 2M KCl, shaking for 1 h, and percolating through filters. The concentrations of ${\text{NO}}_{3}^{-}$−N and ${\text{NH}}_{4}^{+}$−N were determined by continuous flow analytical system (San++System, Skalar, Holland). DOC was determined by carbon nitrogen analyzer (Multi N/C 3000, Analytik Jena, Germany). Total carbon (TC) and total nitrogen (TN) were determined by a carbon nitrogen analyzer (Vario Max CN, Elementar, Germany).

### Soil DNA extraction and purification

Soil DNA was extracted from 0.5 g fresh soil using the Fast DNA® SPIN Kit for soil (MP Bio medicals, Santa Ana, CA) according to the manufacturer's instructions. Then, the soil DNA was eluted in 60 μl DES. Crude DNA was purified by using the Power Clean® Pro DNA Kit (MO BIO, Laboratories, Inc.) and eluted in 35 μl TE buffer. Purified DNA was quantified using the Nano Drop ND-1000 spectrophotometer (Nano Drop Technologies, Wilmington, DE) and stored at −20°C.

### Barcoded pyro-sequencing of bacterial and fungal communities

Amplification, purification, pooling, and pyro-sequencing of V4-V5 hyper-variable regions of the bacterial 16S rRNA genes were performed as described previously (Biddle et al., 2008) and the fungal variable ITS-1 regions were also performed as described previously (Buée et al., 2009). Briefly, primers F519 (5′-CAGCMGCCGC GGTAATWC-3′)/R907 (5′-CCGTCAATTCMTTT RAGTTT-3′) and ITS1-F (5′-CTTGGTCATTTAGA GGAAGTAA-3′)/ITS2 (5′-GCTGCGTTCTTCATCGATGC-3′) were used to amplify bacterial 16S rRNA gene and fungal ITS-1 region, respectively. A unique 7 bp barcode sequence was added to the forward primers to distinguish PCR products of different samples (multiplexing). The PCR reaction was conducted in a 50 μl volume reaction mixture containing 20 μM of forward and reverse primers, respectively, 25 μl of Taq DNA polymerase mix (TaKaRa, Japan), and ~30 ng DNA. Each sample was amplified under the following conditions: 94°C for 5 min, 30 cycle of 94°C for 30 s, 55°C for 30 s, and 72°C for 30 s for bacteria and 30 cycle of 94°C for 45 s, 56°C for 1 min, and 72°C for 90 s for fungi, then a final extension at 72°C for 10 min. PCR products were then purified using the QIAquick PCR Purification kit (QIAGEN, Germany), and quantified by Nano Drop ND-1000 (Thermo Scientific, USA). Equal amount of PCR products were pooled into a single tube and sequenced on a Roche FLX 454 pyro-sequencing machine (Roche Diagnostics Corporation, Branford, CT, USA).

### Processing of pyro-sequencing data

Data were analyzed using the QIIME 1.8.0 pipeline (Caporaso et al., 2010). Briefly, bacterial and fungal sequences were quality trimmed, and the 7 bp barcode was used to assign sequences to soil samples. Zero mismatches were allowed during filtering, and sequences < 200 bp were removed for bacteria; sequences < 150 bp were removed for fungi. Bacterial and fungal raw reads were denoised by QIIME's implementation of denoising (Reeder and Knight, 2010). Chimera checking was performed using UCHIME (Edgar et al., 2011) and sequences were binned into operational taxonomic units (OTUs) using 97% similarity identity with a *de novo* clustering-usearch algorithm. Representative sequences, the most abundant sequences showing up in each OTU, were aligned by PyNAST. The taxonomic identities were determined using RDP classifier (Wang et al., 2007). OTUs containing < 2 reads (singletons) were removed. Each bacterial OTU representative sequence was assigned taxonomy against the Greengenes database gg_13_5 (DeSantis et al., 2006). To compare all of the soils at the same level of sampling effort (subsampling), 2200 16S rRNA gene sequences were randomly selected for alpha- and beta-diversity analyses. Each fungal OTU representative sequence was assigned taxonomy against the UNITE database its_12_11 (Seifert, 2009). To compare all of the soils at the same level of sampling effort, 1900 ITS sequences were randomly selected for alpha- and beta-diversity analyses. The subsampling sequencing depth for each dataset was determined by the minimum number of sequences observed in any one sample. ITS OTUs that were not assigned to fungi were removed before subsampling.

### Statistical analysis

Variables that did not meet the assumptions of parametric statistical tests (normality and homoscedasticity of errors) were log-transformed (${\text{NH}}_{4}^{+}$-N, DOC, relative abundance of Chloroflexi and Firmicutes, relative abundance of Ascomycota, Basidiomycota, and Glomeromycota). Data normality was tested with a Shapiro-Wilk test. Tukey HSD was used for multiple comparisons with a *p* = 0.05 grouping baseline. General Linear Models (GLMs) were used to evaluate the main and interactive effects of warming and altered precipitation. Changes in bacterial and fungi community structure were evaluated by using nonmetric multidimensional scaling (NMDS) based on Bray-Curtis distance, as calculated using the “vegan” package in the R environment. Significant differences among different data sets were determined using PerMANOVA analyses (adonis function). Explanatory relationships between the aggregate environmental factors and microbial community structure were assessed by Mantel test using Spearman's correlation. The effects of warming and altered precipitation on abundance of a given OTU were determined using a response ratio at 95% confidence interval (CI). The 95% CI = *rr*_*i*_ ± 1.96 × $\sqrt{V_{i}}$, *rr*_*i*_ = $\text{ln}\left(\bar{x_{i}}∕\bar{y_{i}}\right)$ (*i* = 1 … *n*), $\bar{x}$ is the mean OTU numbers of the treated sample, $\bar{y}$ is the mean OTU numbers of the control sample and *V*_*i*_ = $\frac{{s_{x_{i}}}^{2}}{m_{x_{i}}{\bar{x_{i}}}^{2}}+\;\frac{{s_{y_{i}}}^{2}}{m_{y_{i}}{\bar{y_{i}}}^{2}}$ (*i* = 1 … *n*), where *s* is the standard deviation of OTUs in treated or control samples, *m* is the number of OTU in treated and control samples. The significance value *RR*_*i*_ = (*rr*_*i*_ + 1.96 $\sqrt{V_{i}}$) × (*rr*_*i*_ − 1.96 $\sqrt{V_{i}}$). When *RR*_*i*_ > 0, the OTU in treated sample is significantly different from control (Luo et al., 2006; Xiang et al., 2015).

Structural equation modeling (SEM; Grace, 2006) was used to analyze interrelationships among climate change stressors, soil biogeochemical variables and soil microorganisms. Predicted causal relationships between variables were based on prior knowledge of effects of climate change on soil microorganisms. Principal Co-ordinates Analysis (PCoA) was used to simplify the variance of microbial community composition and the first principal coordinate axis (PC1) was used to proxy the variance of microbial community composition across different samples (Veen et al., 2010). By stepwise removal of the non-significant paths in the initial model, we selected the final model that best fit our data. The adequacy of the model was determined by χ^2^-test, GIF (Goodness of fit) index, and RMSEA (root mean squared error of approximation) index. The χ^2^-test was employed to test whether the model reasonably explained the patterns in the data and the RMSEA index was used to adjust for sample size. Favorable model fits were suggested by non-significant χ^2^-test (*P* > 0.05), high GIF and low RMSEA (< 0.05). SEM was conducted in Amos 18.0 (IBM, Chicago, IL, USA).

### Nucleotide sequence accession numbers

Sequences were submitted in the NCBI Sequence Read Archive (SRA) under accession number SRP 049447 (www.ncbi.nlm.nih.gov/sra/?term=SRP049447).

## Results

### Effects of warming and altered precipitation on soil biogeochemical properties

With the exception of soil moisture and ${\text{NO}}_{3}^{-}$-N, the other measured soil biogeochemical properties were unaltered after 1-year warming and/or altered precipitation treatments (Tables 1, 2). Soil moisture decreased by 43.8% in DP (decreased precipitation) treatment (*p* < 0.01) and by 33.6% in W (warming) treatment (*p* = 0.01). The decrease in soil moisture was intensified in W × DP treatment (warming coupling with decreased precipitation), where it decreased by 57.8% (*p* < 0.01). Soil ${\text{NO}}_{3}^{-}$-N decreased by 61.6% (*p* = 0.03) in W × DP treatment (Table 1).

**Table 1:** **Soil biogeochemical properties in warming and altered precipitation treatments**.

**Treatment**	**pH**	**Moisture (%)**	**${\text{NH}}_{4}^{+}$-N (mg/kg)**	**${\text{NO}}_{3}^{-}$-N (mg/kg)**	**DOC (mg/kg)**	**TC (%)**	**TN (%)**
Control	7.68 ± 0.13 (a)	56.0 ± 4.2 (a)	21.0 ± 2.3 (a)	47.4 ± 1.5 (a)	736 ± 48 (a)	11.14 ± 0.62 (a)	0.98 ± 0.05 (a)
DP	7.83 ± 0.05 (a)	31.5 ± 4.5 (b)	20.1 ± 1.7 (a)	35.6 ± 7.5 (ab)	682 ± 44 (a)	9.63 ± 0.49 (a)	0.87 ± 0.05 (a)
IP	7.77 ± 0.11 (a)	66.2 ± 3.8 (a)	20.2 ± 2.6 (a)	49.8 ± 5.0 (a)	696 ± 64 (a)	9.25 ± 0.37 (a)	0.91 ± 0.07 (a)
W	7.77 ± 0.07 (a)	37.2 ± 4.2 (b)	19.8 ± 3.9 (a)	45.3 ± 10.5 (ab)	770 ± 126 (a)	10.35 ± 0.67 (a)	0.97 ± 0.03 (a)
W × DP	7.84 ± 0.02 (a)	23.6 ± 1.6 (b)	15.5 ± 1.4 (a)	18.2 ± 4.0 (b)	904 ± 106 (a)	10.00 ± 0.39 (a)	0.87 ± 0.04 (a)
W × IP	7.74 ± 0.14 (a)	64.1 ± 2.5 (a)	25.4 ± 3.2 (a)	54.7 ± 5.4 (a)	822 ± 73 (a)	10.80 ± 0.58 (a)	0.96 ± 0.05 (a)

*Different letters indicate statistical differences between control, warming, and altered precipitation plots using Tukey's HSD for multiple comparisons (p < 0.05, mean ± SE). DOC, dissolved organic carbon; TC, total carbon; TN, total nitrogen. DP, decreased precipitation for 50%; IP, increased precipitation for 50%; W, warming for 2°C; W × DP, warming for 2°C and decreased precipitation for 50%; W × IP, warming for 2°C and increased precipitation for 50%*.

**Table 2:** **General linear model results for the effects of warming and altered precipitation on soil physicochemical properties and soil microbial communities**.

**Treatment**	**warming**		**precipitation**		**Warming × precipitation**	
	**F**	**p**	**F**	**p**	**F**	**p**
**SOIL BIOGEOCHEMICAL PROPERTIES**						
pH	0.12	0.73	0.69	0.51	0.19	0.83
moisture	10.57	**<0.01**	54.23	**<0.01**	2.77	0.08
${\text{NH}}_{4}^{+}$-N	0.01	0.93	1.74	0.19	1.76	0.19
${\text{NO}}_{4}^{-}$-N	0.9	0.35	8.82	**<0.01**	1.64	0.21
DOC	3.56	0.07	0.14	0.87	0.65	0.53
TC	0.76	0.39	1.67	0.21	2.4	0.11
TN	0.17	0.69	2.24	0.13	0.28	0.76
**MICROBIAL DIVERSITY**						
Bacterial richness	0.05	0.83	0.83	0.45	0.67	0.52
Bacterial evenness	0.35	0.56	0.21	0.81	0.56	0.58
Fungal richness	0.01	0.91	1.6	0.23	1.31	0.29
Fungal evenness	0.11	0.74	0.89	0.43	0.43	0.65
**RELATIVE ABUNDANCE OF 15 MOST ABUNDANT LINEAGES**						
Actinobacteria	5.06	**0.04**	2.92	0.08	0.11	0.9
Alphaproteobacteria	0.03	0.87	4.1	**0.03**	0.87	0.43
Betaproteobacteria	5.85	**0.03**	2.25	0.13	1.55	0.24
Bacteroidetes	3.31	0.08	6.78	**0.01**	0.74	0.49
Acidobacteria	0.06	0.81	3.65	**0.04**	1.03	0.38
Chloroflexi	0.43	0.52	1.42	0.26	0.08	0.93
Deltaproteobacteria	0.88	0.36	0.51	0.61	0.29	0.75
Gammaproteobacteria	1.81	0.19	5.74	**0.01**	2.89	0.08
Planctomycetes	0.8	0.38	0.59	0.57	1.59	0.23
Firmicutes	0.94	0.34	0.23	0.8	1.34	0.28
Gemmatimonadetes	0.01	0.93	0.45	0.65	0.59	0.57
Ascomycota	0.03	0.86	0.54	0.59	0.26	0.77
Basidiomycota	0.15	0.71	0.57	0.58	0.11	0.9
Zygomycota	0.83	0.37	0.43	0.66	0.56	0.58
Glomeromycota	1.38	0.26	0.28	0.76	1.51	0.25

*Significant effects (p < 0.05) are given in bold*.

### Effects of warming and altered precipitation on soil bacterial and fungal diversity and community structure

Pyro-sequencing generated a total of 73,904 (range from 2208 to 3553) and 422,903 (range from 1959 to 40,516) quality sequences for bacteria and fungi, respectively. The dominant linkages of bacteria across the alpine grassland soils were Actinobacteria and Alphaproteobacteria, which accounted for ~60% of total sequences (Figure S2A). The dominant phyla of fungi were Ascomycota and Basidiomycota, accounting for ~80% of total sequences (Figure S2B). Richness (i.e., number of observed OTUs) and Simpson evenness (both calculated at the subsampled depth of 2200 and 1900 randomly selected for bacteria and fungi, respectively) were not affected by warming and altered precipitation (Table S1).

**Figure 1:**
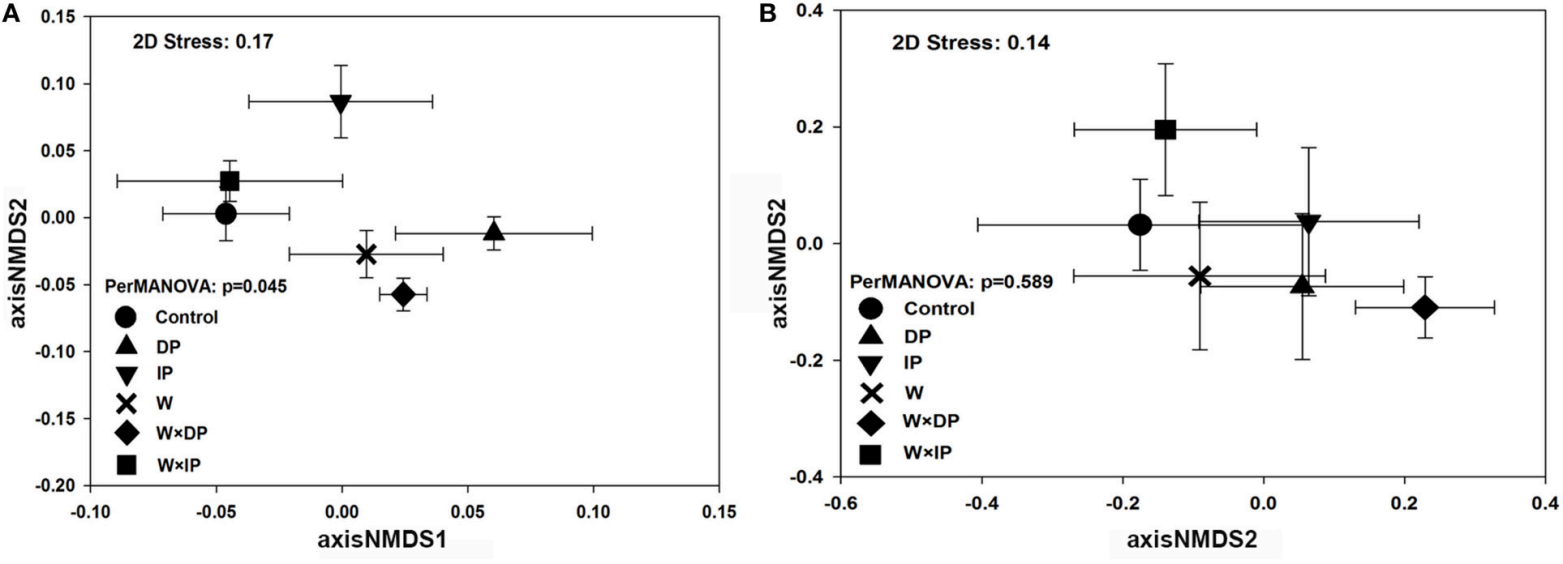
**Nonmetric multiple dimension scaling (NMDS) ordination based on Bray-Curtis dissimilarity matrix shows bacterial (A) and fungal (B) community structure among warming and altered precipitation treatments in the experiment sites**. Error bars represent the standard error of mean coordinates. DP, decreased precipitation for 50%; IP, increased precipitation for 50%; W, warming for 2°C; W × DP: warming for 2°C and decreased precipitation for 50%; W × IP: warming for 2°C and increased precipitation for 50%.

**Figure 2:**
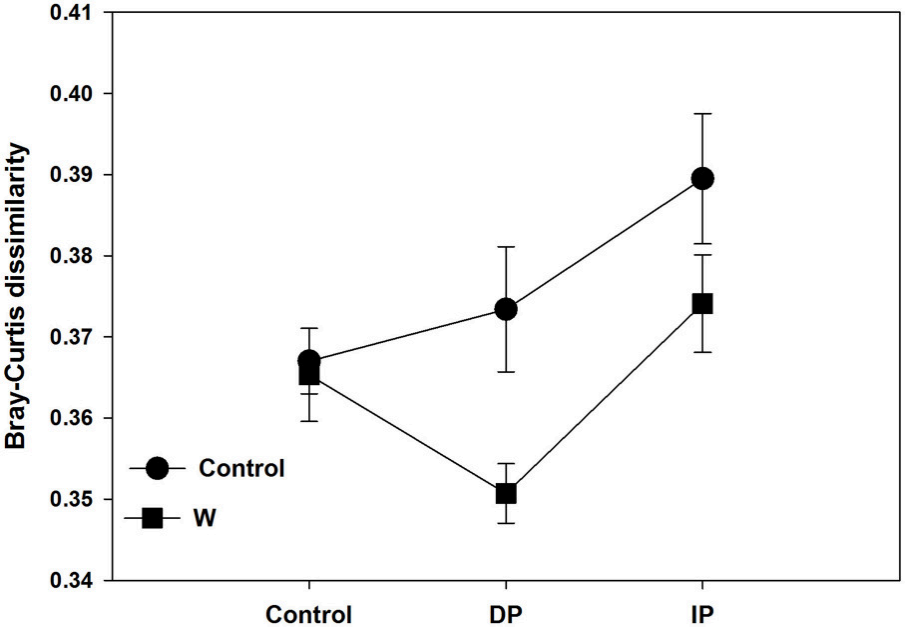
**Bacterial Bray-Curtis dissimilarity along the precipitation gradients in each of the two warming treatments**. DP, decreased precipitation for 50%; IP, increased precipitation for 50%; W, warming for 2°C.

Although there were no significant impacts of climate change on bacterial and fungal alpha-diversity, shifts in bacterial community structure were detected (*p* = 0.045; Figure 1A). Warming coupled with decreased precipitation significantly altered bacterial community structure (PerMANOVA *R*^2^ = 0.14, *p* = 0.03; Table S3) as compared to the control. Bacterial community structure of W × DP plots were also different from that of IP (PerMANOVA *R*^2^ = 0.17, *p* < 0.01; Table S3) and W × IP (PerMANOVA *R*^2^ = 0.17, *p* = 0.01; Table S3) plots. We also examined the main effects and interactions of warming and altered precipitation treatments and the effect of interactions was not significant for bacterial bray-curtis dissimilarity. (Figure 2, Table S2). For the fungal community structure, there was no detectable influence of short-term warming and altered precipitation (*p* = 0.59; Figure 1B).

### Effects of warming and altered precipitation on the dominant bacterial taxa

**Figure 3:**
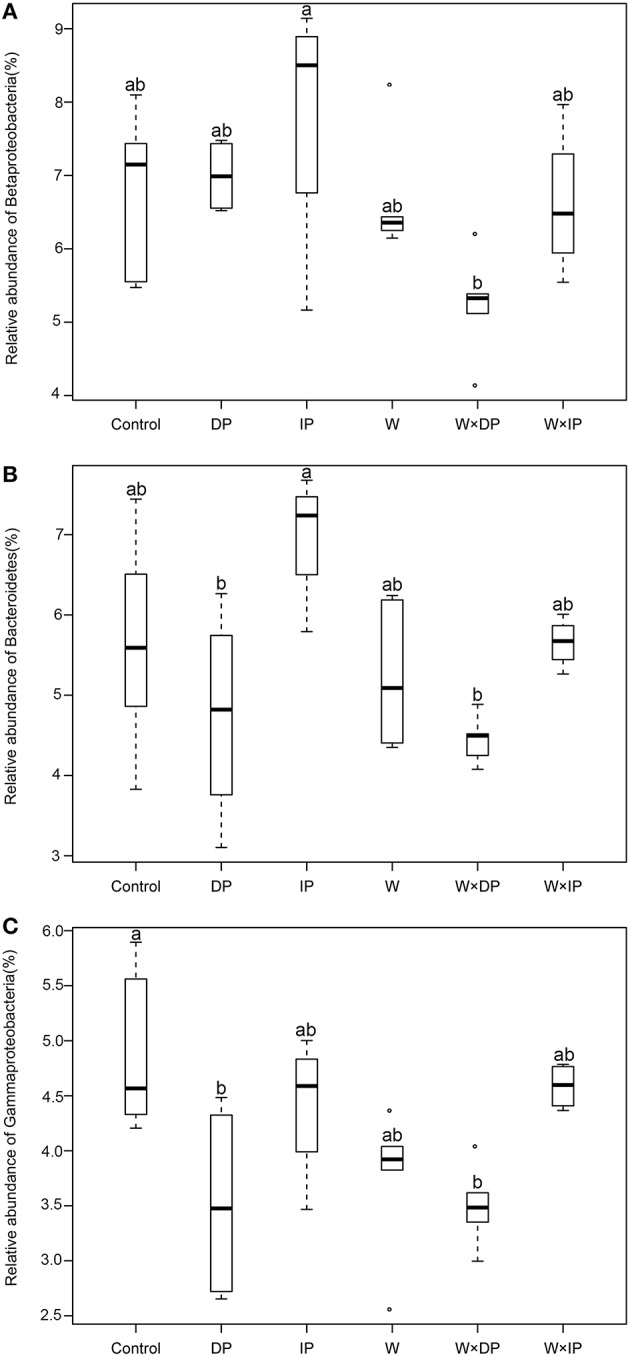
**Relative abundance of Betaproteobacteria (A), Bacteroidetes (B), and Gammaproteobacteria (C) under warming and altered precipitation treatments**. Different letters indicate statistical differences between control, warming, and altered precipitation plots using Tukey's HSD for multiple comparisons (*p* < 0.05, mean ± SE). DP, decreased precipitation for 50%; IP, increased precipitation for 50%; W, warming for 2°C; W × DP, warming for 2°C and decreased precipitation for 50%; W × IP, warming for 2°C and increased precipitation for 50%.

**Figure 4:**
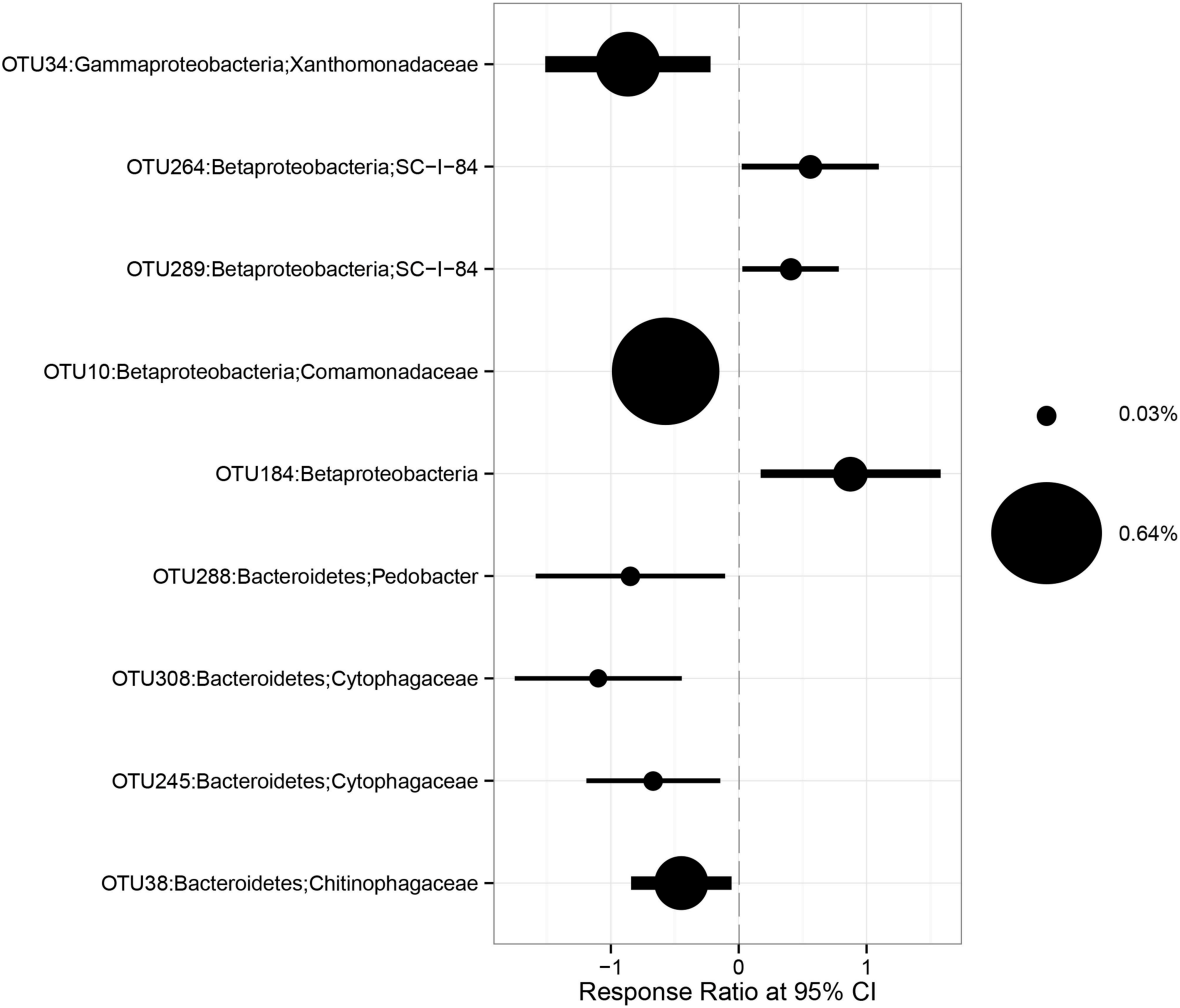
**Response ratio method of changes in abundance of OTUs belonging to Bacteroidetes, Betaproteobacteria, and Gammaproteobacteria in W × DP treatment (warming coupling with decreased precipitation) relative to the control**.

The relative abundances of some bacterial taxa were changed after 1-year warming and altered precipitation treatments. For example, compared with IP plots, the relative abundances of Betaproteobacteria and Bacteroidetes decreased by 33.1% (*p* = 0.02) and 36.4% (*p* < 0.01) in W × DP plots (Figures 3A,B). The relative abundance of Gammaproteobacteria was lower in both DP (*p* = 0.04) and W × DP plots (*p* = 0.03, Figure 3C) than in control plots. At the OTU level (97% 16S rRNA identity), all responsive OTUs of Gammaproteobacteria were decreased in DP and W × DP treatment compared with control (Figure 4, Figure S3). In contrast, dominant fungal taxa were not affected by warming and altered rainfall after 1-year (Table 2).

### Effects of environmental variables on soil microbial community composition

**Figure 5:**
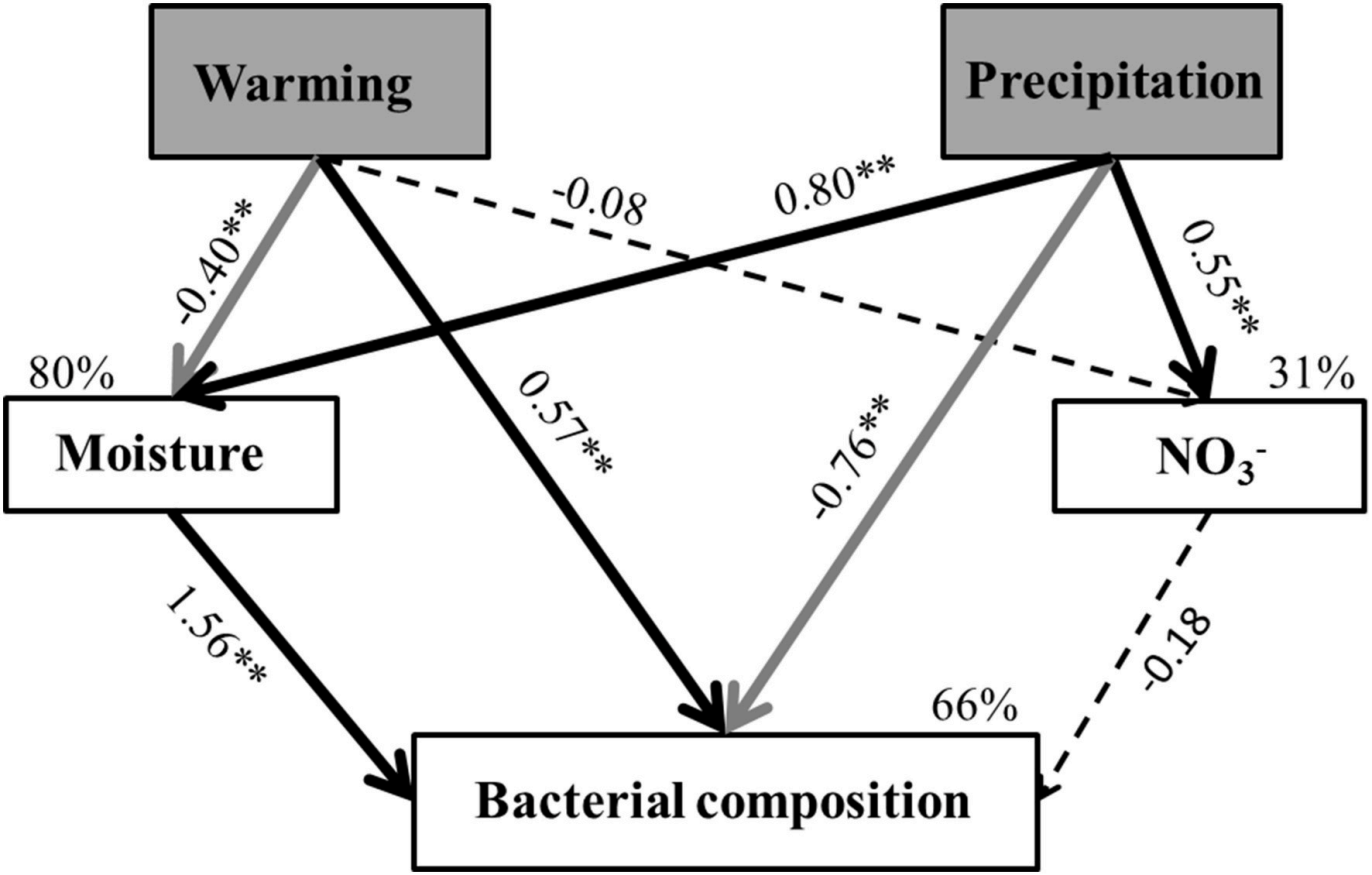
**Structural equation model shows the effects of warming and altered precipitation on soil bacterial composition**. Causal influences of warming and altered precipitation (exogenous variable; gray rectangle) on soil ${\text{NO}}_{3}^{-}$, soil moisture and bacterial community composition (endogenous variables; white rectangle). The model fit the data well: χ^2^ = 1.22, *df* = 2, *P* = 0.54, GIF = 0.98, RMSEA < 0.001 Numbers on arrows are standardized path coefficients (equivalent to correlation coefficients), asterisks followed the numbers means significant relationships (^**^p < 0.01 and ^*^p < 0.05). Dark solid arrows indicate significant positive relationships, gray solid arrows indicate significant negative relationships and black dash arrows indicate non-significant path coefficients (*p* > 0.05). Width of the arrows shows the strength of the causal relationship. Percentages (*R*^2^) close to endogenous variables indicate the variance explained by the climatic and soil factors.

The SEM adequately fitted the soil bacterial composition data (χ^2^ = 1.22, *df* = 2, *P* = 0.54; GIF = 0.98; RMSEA < 0.01; standardized path coefficients were given in Figure 5). The final model explained ~31% of the variance of ${\text{NO}}_{3}^{-}$, ~80% in the soil moisture content, 66% for bacterial community composition.

Consistent with the ANOVA results (Table 1), warming and altered precipitation treatments affected soil moisture content (*p* < 0.01, Figure 5). In the soil bacterial community composition model, bacterial composition was driven by direct impacts of warming and altered precipitation, and was positively related to soil moisture. Furthermore, the Mantel test also detected that bacterial community composition were significantly correlated with soil moisture (Mantel *r* = 0.31, *p* < 0.01; Table S4). We also found that the relative abundances of Bacteroidetes (*p* < 0.01) and Gammaproteobacteria (*p* < 0.01) had strong correlation with soil moisture, while Betaproteobacteria had no significant correlation (*p* = 0.07) with soil moisture (Table S5).

## Discussion

Soil microorganisms play an important role in carbon and nitrogen cycling, which influence ecosystem functioning. However, the inconsistencies across climate change studies, including the types of ecosystems investigated and the techniques used, make it difficult to understand exactly how soil microbes respond to climate change drivers, especially in the case of multifactor climate changes stressors. We used a short-term *in situ* manipulation of warming and altered precipitation to investigate how warming, altered precipitation and their interaction affected both abundance and composition of soil bacterial and fungal communities in an ecosystem particularly sensitive to climate changes. After 1-year of treatment, the alpha-diversity of bacteria and fungi were not affected by warming and altered precipitation. However, bacterial community structure was sensitive in the near-term. We observed changes in the relative abundance of particular bacterial taxa, and were able to attribute these changes to the main experimental effects of warming or altered precipitation.

Climate change has the potential to affect soil microbial community structure by selecting species that can adapt to rapid changes in the environments (Fierer et al., 2003; Gray et al., 2011), and this may affect the function of ecosystem in the future. In this study, major shifts in bacterial community were observed in some specific dominant taxa (Figures 3, 4, Figure S3) which occupied unique niche. The relative abundance of Gammaproteobacteria (dominant order is Xanthomonadales) was significantly lower in DP and W × DP plots compared with the control, and positively correlated with soil moisture (Table S5). The relative abundance of Bacteroidetes [dominant order are Cytophagales and (Saprospirales)] was also strongly influenced by soil moisture. These two bacterial phyla and subphyla were observed increasing after rainfall addition treatment in a California grassland (Cruz-Martínez et al., 2009), and they were mainly affected by the main effect of precipitation in our study (Table 2). On the other hand, our results showed that the relative abundance of Betaproteobacteria (dominant order is Burkholderiales) was strongly impacted by the main effect of warming, and this subphylum has been reported decreasing in relative abundance when summer warming happening in sub-arctic peat bog (Weedon et al., 2012). These groups contain members that are copiotrophic, with an adaptive r-selected strategy to drive their rapid response to resource availability (Fierer et al., 2007). But the r-K spectrum of lifestyle among microbial community members has been proposed to be a key factor that may help to explain the response of community structure to climate change stressors (de Vries and Shade, 2013). Together, our results suggest that copiotrophic microbes are sensitive in response to warming and altered precipitation in alpine grassland.

Warming coupled with decreased precipitation significantly impacted soil bacterial community structure, and those changes were also affected by fluctuations in soil moisture (Figure 5, Table S4). A previous study conducted in a tall grass prairie showed that warming combined with drought significantly reduced soil moisture, which resulted in a decrease in microbial abundance (Sheik et al., 2011). Relatedly, studies of three different ecosystems (arctic tundra, grassland, and savannah) that were exposed to warming and altered precipitation manipulation found that soil proteolytic enzyme activity was suppressed by decreased soil moisture (Brzostek et al., 2012). Our study and these together suggest that changes in soil moisture may play an important role in microbial responses to warming and altered precipitation. It may be expected that certain microorganisms may be more resistant to soil moisture changes than others; for instance, bacteria with special adaptive traits to desiccation, such as thickened cell walls, may be able to tolerate moisture fluctuation while others may not (Schimel et al., 2007; Manzoni et al., 2014). Soil moisture has been shown to be a critical factor affecting microbial community structure. For example, wet soils with high water content have lower soil pore connectivity, which limits diffusion and control the redox state to anaerobic (Manzoni et al., 2012). On the other hand, dry soils with low water content have more restricted nutrient diffusion (Bouskill et al., 2012), which affects microbial access to substrate. This may lead to shifts in soil microbial community structure because some members are sensitive to low water content because of these and other interacting moisture-dependent processes.

Compared with bacterial community composition, the fungal community composition was statistically indistinguishable among warming and altered precipitation treatments. Morphology and physiological characters may help fungi to overcome the short-term stressors. When water is limited, fungal hyphal networks would be forced to expand so that they can access more nutrients and moisture (Manzoni et al., 2012). Species with wide environmental tolerance ranges may persist in the fungal community (Hawkes et al., 2011) and they may play a role in moderating community response to short-term warming and altered precipitation. Furthermore, shifts in the plant community may also modulate fungal response to climate change because these communities are closely intertwined. For example, in three different tropical rainforests, Peay et al. (2013) found strong correlations between plant and fungal community structure. A previous study also indicated that increased sedges and reduced grasses led to low abundance of arbuscular mycorrhizal fungi (Rudgers et al., 2014) after warming. In the absence of an altered plant community (Figure S4), we may expect limited response of their closely associated fungi in alpine grassland.

The responses of microbial communities to climate changes likely have consequences for ecosystem functioning, and sensitive ecosystem, like the Tibetan plateau, can provide insights into these consequences. A recent study in the Tibetan grassland showed that warming decreased microbial capacity to decompose recalcitrant carbon, which ameliorated soil carbon loss and provided an explanation as to the observed relatively stable carbon stock in Tibet (Yue et al., 2015). Interestingly, a large-scale investigation in the Tibetan plateau alpine grassland showed that spatial distribution of topsoil organic carbon stock had positive correlation with soil moisture induced by natural precipitation gradient (Yang et al., 2008), though the underlying mechanisms remain elusive. Considering the pivotal role of microbial communities on mediating terrestrial carbon balance (Nie et al., 2013), below-ground micro-communities were expected to play an essential role. Some bacterial taxa have previously been found to have positive relationships with soil available carbon (Fierer et al., 2007; Eilers et al., 2010, 2012). In our study, we found the relative abundances of Bacteroidetes and Gammaproteobacteria have a positive relationship with soil moisture (Table S5), which may provide some linkages explaining for the positive relationship between soil organic carbon and soil moisture. The strong correlation between soil carbon, soil microorganisms, and soil water availability suggests that certain bacterial taxa that are selected by moisture regime may serve as early indicators of climate change, and are likely linked with carbon-climate feedback. However, in our study, there was no significant change in soil dissolved organic carbon after 1-year warming and altered precipitation treatments. Further, investigation over longer timescales will be necessary to determine the explicit relationships between soil microbial communities and soil carbon in response to multifactor climate changes.

## Author contributions

HC, JH, and YS designed experiments; KZ carried out experiments; KZ, RS, and XJ analyzed experimental results; KZ wrote the manuscript; HC, YS, XJ, JH, RS, YY, AS assisted with revising the manuscript.

### Conflict of interest statement

The authors declare that the research was conducted in the absence of any commercial or financial relationships that could be construed as a potential conflict of interest.

## References

[B1] Bellard C. Bertelsmeier C. Leadley P. Thuiller W. Courchamp F. (2012). Impacts of climate change on the future of biodiversity. Ecol. Lett. 15, 365–377. 10.1111/j.1461-0248.2011.01736.x 22257223 PMC3880584

[B2] Biddle J. F. Fitz-Gibbon S. Schuster S. C. Brenchley J. E. House C. H. (2008). Metagenomic signatures of the Peru Margin subseafloor biosphere show a genetically distinct environment. Proc. Natl. Acad. Sci. U.S.A. 105, 10583–10588. 10.1073/pnas.0709942105 18650394 PMC2492506

[B3] Bouskill N. J. Lim H. C. Borglin S. Salve R. Wood T. E. Silver W. L.. (2012). Pre-exposure to drought increases the resistance of tropical forest soil bacterial communities to extended drought. ISME J. 7, 384–394. 10.1038/ismej.2012.113 23151641 PMC3554394

[B4] Brzostek E. R. Blair J. M. Dukes J. S. Frey S. D. Hobbie S. E. Melillo J. M. (2012). The effect of experimental warming and precipitation change on proteolytic enzyme activity: positive feedbacks to nitrogen availability are not universal. Glob. Change Biol. 18, 2617–2625. 10.1111/j.1365-2486.2012.02685.x

[B5] Buée M. Reich M. Murat C. Morin E. Nilsson R. H. Uroz S. (2009). 454 Pyrosequencing analyses of forest soil reveal an unexpectedly high fungal diversity. New Phytol. 184, 449–456. 10.1111/j.1469-8137.2009.03003.x 19703112

[B6] Caporaso J. G. Kuczynski J. Stombaugh J. Bittinger K. Bushman F. D. Costello E. K.. (2010). QIIME allows analysis of high-throughput community sequencing data. Nat. Methods 7, 335–336. 10.1038/nmeth.f.303 20383131 PMC3156573

[B7] Chen H. Zhu Q. Peng C. H. Wu N. Wang Y. F. Fang X. Q.. (2013). The impacts of climate change and human activities on biogeochemical cycles on the Qinghai-Tibetan Plateau. Glob. Change Biol. 19, 2940–2955. 10.1111/gcb.12277 23744573

[B8] Contosta A. R. Frey S. D. Cooper A. B. (2015). Soil microbial communities vary as much over time as with chronic warming and nitrogen additions. Soil Biol. Biochem. 88, 19–24. 10.1016/j.soilbio.2015.04.013

[B9] Cruz-Martínez K. Suttle K. B. Brodie E. L. Power M. E. Andersen G. L. Banfield J. F. (2009). Despite strong seasonal responses, soil microbial consortia are more resilient to long-term changes in rainfall than overlying grassland. ISME J. 3, 738–744. 10.1016/j.soilbio.2015.04.013 19279669

[B10] DeAngelis K. M. Pold G. Topçuoğlu B. D. van Diepen L. T. Varney R. M. Blanchard J. L.. (2015). Long-term forest soil warming alters microbial communities in temperate forest soils. Front. Microbiol. 6:104. 10.3389/fmicb.2015.00104 25762989 PMC4327730

[B11] DeSantis T. Z. Hugenholtz P. Larsen N. Rojas M. Brodie E. L. Keller K.. (2006). Greengenes, a chimera-checked 16S rRNA gene database and workbench compatible with ARB. Appl. Environ. Microb. 72, 5069–5072. 10.1128/AEM.03006-05 16820507 PMC1489311

[B12] de Vries F. T. Shade A. (2013). Controls on soil microbial community stability under climate change. Front. Microbiol. 4:265. 10.3389/fmicb.2013.00265 24032030 PMC3768296

[B13] Edgar R. C. Haas B. J. Clemente J. C. Quince C. Knight R. (2011). UCHIME improves sensitivity and speed of chimera detection. Bioinformatics 27, 2194–2200. 10.1093/bioinformatics/btr381 21700674 PMC3150044

[B14] Eilers K. G. Debenport S. Anderson S. Fierer N. (2012). Digging deeper to find unique microbial communities: the strong effect of depth on the structure of bacterial and archaeal communities in soil. Soil Biol. Biochem. 50, 58–65. 10.1016/j.soilbio.2012.03.011

[B15] Eilers K. G. Lauber C. L. Knight R. Fierer N. (2010). Shifts in bacterial community structure associated with inputs of low molecular weight carbon compounds to soil. Soil Biol. Biochem. 42, 896–903. 10.1016/j.soilbio.2010.02.003

[B16] Evans S. E. Wallenstein M. D. Burke I. C. (2014). Is bacterial moisture niche a good predictor of shifts in community composition under long-term drought? Ecology 95, 110–122. 10.1890/13-0500.1 24649651

[B17] Fierer N. Bradford M. A. Jackson R. B. (2007). Toward an ecological classification of soil bacteria. Ecology 88, 1354–1364. 10.1890/05-1839 17601128

[B18] Fierer N. Schimel J. P. Holden P. A. (2003). Influence of drying-rewetting frequency on soil bacterial community structure. Microb. Ecol. 45, 63–71. 10.1007/s00248-002-1007-2 12469245

[B19] Grace J. B. (2006). Structural Equation Modeling and Natural Systems. Cambridge: Cambridge University Press.

[B20] Gray S. B. Classen A. T. Kardol P. Yermakov Z. Michael Mille R. (2011). Multiple climate change factors interact to alter soil microbial community structure in an old-field ecosystem. Soil Sci. Soc. Am. J. 75, 2217–2226. 10.2136/sssaj2011.0135

[B21] Hawkes C. V. Kivlin S. N. Rocca J. D. Huguet V. Thomsen M. A. Suttle K. B. (2011). Fungal community responses to precipitation. Glob. Change Biol. 17, 1637–1645. 10.1111/j.1365-2486.2010.02327.x

[B22] Hoeppner S. S. Dukes J. S. (2012). Interactive responses of old-field plant growth and composition to warming and precipitation. Glob. Change Biol. 18, 1754–1768. 10.1111/j.1365-2486.2011.02626.x

[B23] Horz H. Barbrook A. Field C. B. Bohannan B. J. (2004). Ammonia-oxidizing bacteria respond to multifactorial global change. Proc. Natl. Acad. Sci. U.S.A. 101, 15136–15141. 10.1073/pnas.0406616101 15469911 PMC524064

[B24] IPCC (2013). The Physical Science Basis. Contribution of Working Group I to the Fifth Assessment Report of the Intergovernmental Panel on Climate Change Cambridge; New York, NY.

[B25] IUSS Working Group WRB (2007). World Reference Base for Soil Resources 2006, First Update 2007. World Soil Resources Reports No. 103, FAO, Rome.

[B26] Kimball B. (2005). Theory and performance of an infrared heater for ecosystem warming. Glob. Change Biol. 11, 2041–2056. 10.1111/j.1365-2486.2005.1028.x

[B27] Lok C. (2015). Mining the microbial dark matter. Nature 522, 270–273. 10.1038/522270a 26085253

[B28] Luo C. Y. Xu G. P. Chao Z. G. Wang S. P. Lin X. W. Hu Y. G. (2010). Effect of warming and grazing on litter mass loss and temperature sensitivity of litter and dung mass loss on the Tibetan plateau. Glob. Change Biol. 16, 1606–1617. 10.1111/j.1365-2486.2009.02026.x

[B29] Luo Y. Hui D. Zhang D. (2006). Elevated CO2 stimulates net accumulations of carbon and nitrogen in land ecosystems: a meta-analysis. Ecology 87, 53–63. 10.1890/04-1724 16634296

[B30] Luo Y. Q. Gerten D. Le Maire G. Parton W. J. Weng E. Zhou X. H. (2008). Modeled interactive effects of precipitation, temperature, and [CO2] on ecosystem carbon and water dynamics in different climatic zones. Glob. Change Biol. 14, 1986–1999. 10.1111/j.1365-2486.2008.01629.x

[B31] Luo Y. Q. Wan S. Q. Hui D. F. Wallace L. L. (2001). Acclimatization of soil respiration to warming in a tall grass prairie. Nature 413, 622–625. 10.1038/35098065 11675783

[B32] Manzoni S. Schaeffer S. M. Katul G. Porporato A. Schimel J. P. (2014). A theoretical analysis of microbial eco-physiological and diffusion limitations to carbon cycling in drying soils. Soil Biol. Biochem. 73, 69–83. 10.1016/j.soilbio.2014.02.008

[B33] Manzoni S. Schimel J. P. Porporato A. (2012). Responses of soil microbial communities to water stress: results from a meta-analysis. Ecology 93, 930–938. 10.1890/11-0026.1 22690643

[B34] Nie M. Pendall E. Bell C. Gasch C. K. Raut S. Tamang S.. (2013). Positive climate feedbacks of soil microbial communities in a semi-arid grassland. Ecol. Lett. 16, 234–241. 10.1111/ele.12034 23157642

[B35] Peay K. G. Baraloto C. Fine P. V. (2013). Strong coupling of plant and fungal community structure across western Amazonian rainforests. ISME J. 7, 1852–1861. 10.1038/ismej.2013.66 23598789 PMC3749505

[B36] Reeder J. Knight R. (2010). Rapidly denoising pyrosequencing amplicon reads by exploiting rank-abundance distributions. Nat. Methods 7, 668–669. 10.1038/nmeth0910-668b 20805793 PMC2945879

[B37] Rinnan R. Michelsen A. Bååth E. Jonasson S. (2007). Fifteen years of climate change manipulations alter soil microbial communities in a subarctic heath ecosystem. Glob. Change Biol. 13, 28–39. 10.1111/j.1365-2486.2006.01263.x

[B38] Rudgers J. A. Kivlin S. N. Whitney K. D. Price M. V. Waser N. M. Harte J. (2014). Responses of high-altitude graminoids and soil fungi to 20 years of experimental warming. Ecology 95, 1918–1928. 10.1890/13-1454.1 25163124

[B39] Rui J. P. Li J. B. Wang S. P. An J. X. Liu W. Lin Q. Y.. (2015). Responses of bacterial communities to simulated climate changes in Alpine meadow soil of the Qinghai-Tibet Plateau. Appl. Environ. Microb. 81, 6070–6077. 10.1128/AEM.00557-15 26116682 PMC4551261

[B40] Schimel J. Balser T. C. Wallenstein M. (2007). Microbial stress-response physiology and its implications for ecosystem function. Ecology 88, 1386–1394. 10.1890/06-0219 17601131

[B41] Seifert K. A. (2009). Progress towards DNA barcoding of fungi. Mol. Ecol. Resour. 9, 83–89. 10.1111/j.1755-0998.2009.02635.x 21564968

[B42] Sheik C. S. Beasley W. H. Elshahed M. S. Zhou X. H. Luo Y. Q. Krumholz L. R. (2011). Effect of warming and drought on grassland microbial communities. ISME J. 5, 1692–1700. 10.1038/ismej.2011.32 21451582 PMC3176507

[B43] Singh B. K. Bardgett R. D. Smith P. Reay D. S. (2010). Microorganisms and climate change: terrestrial feedbacks and mitigation options. Nat. Rev. Microbiol. 8, 779–790. 10.1038/nrmicro2439 20948551

[B44] Suseela V. Tharayil N. Xing B. S. Dukes J. S. (2014). Warming alters potential enzyme activity but precipitation regulates chemical transformations in grass litter exposed to simulated climatic changes. Soil Biol. Biochem. 75, 102–112. 10.1016/j.soilbio.2014.03.022

[B45] Veen G. F. Olff H. Duyts H. Van Der Putten W. H. (2010). Vertebrate herbivores influence soil nematodes by modifying plant communities. Ecology 91, 828–835. 10.1890/09-0134.1 20426340

[B46] Wang Q. Garrity G. M. Tiedje J. M. Cole J. R. (2007). Naive Bayesian classifier for rapid assignment of rRNA sequences into the new bacterial taxonomy. Appl. Environ. Microb. 73, 5261–5267. 10.1128/AEM.00062-07 17586664 PMC1950982

[B47] Wang Y. H. Liu H. Y. Chung H. Yu L. F. Mi Z. R. Geng Y. (2014). Non-growing-season soil respiration is controlled by freezing and thawing processes in the summer monsoon-dominated Tibetan alpine grassland. Glob. Biogeochem. Cycles 28, 1081–1095. 10.1002/2013GB004760

[B48] Weedon J. T. Kowalchuk G. A. Aerts R. van Hal J. van Logtestijn R. Taş N.. (2012). Summer warming accelerates sub-arctic peatland nitrogen cycling without changing enzyme pools or microbial community structure. Glob. Change Biol. 18, 138–150. 10.1111/j.1365-2486.2011.02548.x27337902

[B49] Williams M. A. (2007). Response of microbial communities to water stress in irrigated and drought-prone tallgrass prairie soils. Soil Biol. Biochem. 39, 2750–2757. 10.1016/j.soilbio.2007.05.025

[B50] Xiang X. Gibbons S. M. Yang J. Kong J. Sun R. Chu H. (2015). Arbuscular mycorrhizal fungal communities show low resistance and high resilience to wildfire disturbance. Plant Soil 397, 347–356. 10.1007/s11104-015-2633-z

[B51] Xiong J. B. Sun H. B. Peng F. Zhang H. Y. Xue X. Gibbons S. M.. (2014). Characterizing changes in soil bacterial community structure in response to short-term warming. FEMS Microbiol. Ecol. 89, 281–292. 10.1007/s11104-015-2633-z 24476229

[B52] Xue K. Yuan M. M. Shi Z. J. Qin Y. Deng Y. Cheng L. (2016). Tundra soil carbon is vulnerable to rapid microbial decomposition under climate warming. Nat. Clim. Change 6, 595–600. 10.1038/nclimate2940

[B53] Yang Y. F. Gao Y. Wang S. P. Xu D. P. Yu H. Wu L. W.. (2014). The microbial gene diversity along an elevation gradient of the Tibetan grassland. ISME J. 8, 430–440. 10.1038/ismej.2013.146 23985745 PMC3906809

[B54] Yang Y. H. Fang J. Y. Tang Y. H. Ji C. J. Zheng C. Y. He J. S. (2008). Storage, patterns and controls of soil organic carbon in the Tibetan grasslands. Glob. Change Biol. 14, 1592–1599. 10.1111/j.1365-2486.2008.01591.x

[B55] You Q. L. Kang S. C. Aguilar E. Yan Y. P. (2008). Changes in daily climate extremes in the eastern and central Tibetan Plateau during 1961–2005. J. Geophys. Res. 113:D07101 10.1029/2007JD009389

[B56] Yue H. W. Wang M. M. Wang S. P. Gilbert J. A. Sun X. Wu L. W.. (2015). The microbe-mediated mechanisms affecting topsoil carbon stock in Tibetan grasslands. ISME J. 9, 2012–2020. 10.1038/ismej.2015.19 25689025 PMC4542033

[B57] Zhang B. Chen S. Y. Zhang J. F. He X. Y. Liu W. J. Zhao Q. (2015). Depth-related responses of soil microbial communities to experimental warming in an alpine meadow on the Qinghai-Tibet Plateau. Eur. J. Soil Sci. 66, 496–504. 10.1038/ismej.2015.19

[B58] Zhang N. Wan S. Guo J. Han G. Gutknecht J. Schmid B. (2015). Precipitation modifies the effects of warming and nitrogen addition on soil microbial communities in northern Chinese grasslands. Soil Biol. Biochem. 89, 12–23. 10.1016/j.soilbio.2015.06.022

[B59] Zhang W. Parker K. M. Luo Y. Q. Wan S. Wallace L. L. Hu S. (2005). Soil microbial responses to experimental warming and clipping in a tallgrass prairie. Glob. Change Biol. 11, 266–277. 10.1111/j.1365-2486.2005.00902.x

[B60] Zhang X. M. Zhang G. G. Chen Q. S. Han X. G. (2013). Soil bacterial communities respond to climate changes in a temperate steppe. PLoS ONE 8:e78616. 10.1371/journal.pone.0078616 24250803 PMC3826739

