# Corrigendum: Effects of Short-Term Warming and Altered Precipitation on Soil Microbial Communities in Alpine Grassland of the Tibetan Plateau

**DOI:** 10.3389/fmicb.2017.00667

**Published:** 2017-04-18

**Authors:** Kaoping Zhang, Yu Shi, Xin Jing, Jin-Sheng He, Ruibo Sun, Yunfeng Yang, Ashley Shade, Haiyan Chu

**Affiliations:** ^1^State Key Laboratory of Soil and Sustainable Agriculture, Institute of Soil Science, University of Chinese Academy of SciencesNanjing, China; ^2^University of Chinese Academy of SciencesBeijing, China; ^3^Department of Ecology, College of Urban and Environmental Sciences and Key Laboratory for Earth Surface Processes of the Ministry of Education, Peking UniversityBeijing, China; ^4^Key Laboratory of Adaptation and Evolution of Plateau Biota, Northwest Institute of Plateau Biology, Chinese Academy of SciencesXining, China; ^5^State Key Joint Laboratory of Environment Simulation and Pollution Control, School of Environment, Tsinghua UniversityBeijing, China; ^6^Department of Microbiology and Molecular Genetics, Michigan State UniversityEast Lansing, MI, USA

**Keywords:** climate change, alpine grassland, soil microbial community structure, soil moisture, pyrosequencing

In the published article, there was an error regarding the affiliations for Kaoping Zhang and Ruibo Sun. As well as having Affiliation 1, they should also have the University of Chinese Academy of Sciences, Beijing, China. The authors apologize for this error and state that this does not change the scientific conclusions of the article in any way.

## Conflict of interest statement

The authors declare that the research was conducted in the absence of any commercial or financial relationships that could be construed as a potential conflict of interest.

