# Supplementary material for: Effects of Short-Term Warming and Altered Precipitation on Soil Microbial Communities in Alpine Grassland of the Tibetan Plateau
Source: Front Microbiol. 2016 Jun 30;7:1032. doi: 10.3389/fmicb.2016.01032 (PMC4927576; doi:10.3389/fmicb.2016.01032)

Table S1: Results of soil bacterial and fungal diversity in warming and altered precipitation treatments. Values represent the means of four to five independent replicates per experiment ±the standard errors. DP: decreased precipitation for 50%; IP: increased precipitation for 50%; W: warming for 2 ℃; W×DP: warming for 2 ℃ and decreased precipitation for 50%; W×IP: warming for 2 ℃ and increased precipitation for 50%.

Treatment	Bacteria		fungi	
	richness (No.OTUs)	Simpson evenness	richness (No.OTUs)	Simpson evenness
Control	601±9(a)	0.20±0.01(a)	244±16(a)	0.09±0.03(a)
DP	618±23(a)	0.21±0.02(a)	275±8(a)	0.11±0.02(a)
IP	626±12.(a)	0.21±0.01(a)	263±7(a)	0.13±0.01(a)
W	608±12(a)	0.21±0.02(a)	255±12(a)	0.13±0.03(a)
W×DP	625±13(a)	0.21±0.01(a)	255±10(a)	0.10±0.01(a)
W×IP	604±12(a)	0.20±0.01(a)	270±8(a)	0.13±0.03(a)

Table S2: Results of General Linear Model to explain the variation of soil bacterial community structure. Significant p values are in bold. MS: mean squares; *df*: degrees of freedom.

	Bray-Curtis dissimilarity			
	df	MS	F	p
Warming	1	0.002	8.03	**0.007**
Precipitation	2	0.001	5.75	**0.006**
Warming × precipitation	2	<0.001	1.99	0.149

Table S3: PerMANOVA (pairwise comparisons between treatments) to evaluate variations in the bacterial community structure. Significant p values are in bold. DP: decreased precipitation for 50%; IP: increased precipitation for 50%; W: warming for 2 ℃; W×DP: warming for 2 ℃ and decreased precipitation for 50%; W×IP: warming for 2 ℃ and increased precipitation for 50%.

	PerMANOVA	
Treatment sets	R-Square	p-value
DP vs Control	0.14	0.10
IP vs Control	0.12	0.53
W vs Control	0.13	0.10
W×DP vs Control	0.14	**0.03**
W×IP vs Control	0.11	0.87
IP vs DP	0.16	0.10
W vs DP	0.11	0.88
W×DP vs DP	0.12	0.62
W×IP vs DP	0.16	0.11
W vs IP	0.15	0.12
W×DP vs IP	0.17	**<0.01**
W×IP vs IP	0.13	0.98
W×DP vs W	0.12	0.20
W×IP vs W	0.14	0.13
W×IP vs W×DP	0.17	**0.01**

Table S4: Mantel test (based on Spearman correlation coefficient) between soil physiochemical properties and bacterial community dissimilarity (Bray-Curtis dissimilarity matrix) in warming and altered precipitation treatments. DOC: dissolved organic carbon; TC: total carbon; TN: total nitrogen.

Variables	r	p
moisture	0.31	0.001
NO_3^--N	0.05	0.258
pH	0.32	0.002
TN	0.23	0.005
TC	0.22	0.004
NH_4^+-N	-0.04	0.613
DOC	0.05	0.317

Table S5: Spearman correlation of soil moisture with three bacterial lineages.

Spearman correlation	Betaproteobacteria		Bacteroidetes		Gammaproteobacteria	
	r	p	r	p	r	p
Soil moisture	0.35	0.07	0.64	<0.01	0.74	<0.01

Figure S1: Schematic drawing of the mimic climate change manipulation (Left) and photograph of a experimental plot (Right).

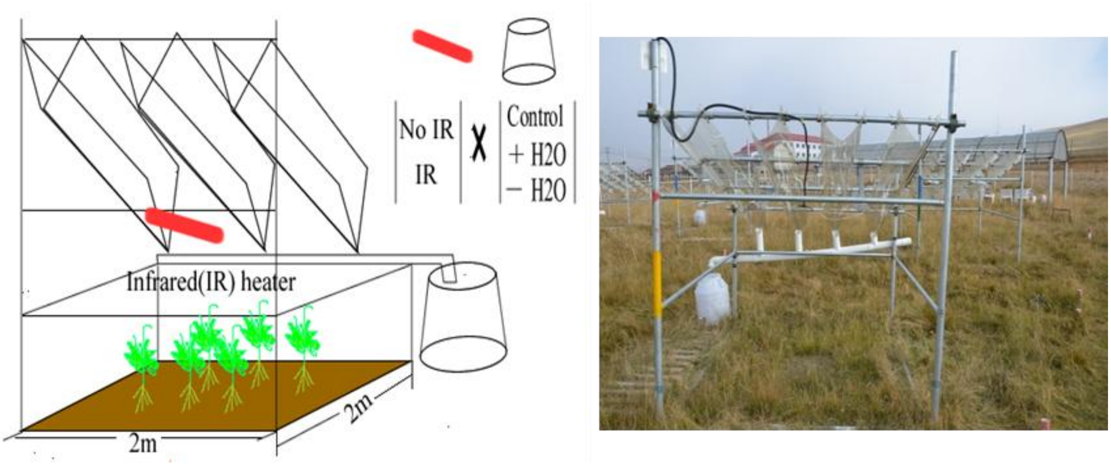

Figure S2: The relative abundance of bacterial taxon (relative abundance >=1%) (A) and relative abundance of fungal dominant taxon (relative abundance >=1%) (B) in six treatments. DP: decreased precipitation for 50%; IP: increased precipitation for 50%; W: warming for 2 ℃; W×DP: warming for 2 ℃ and decreased precipitation for 50%; W×IP: warming for 2 ℃ and increased precipitation for 50%.

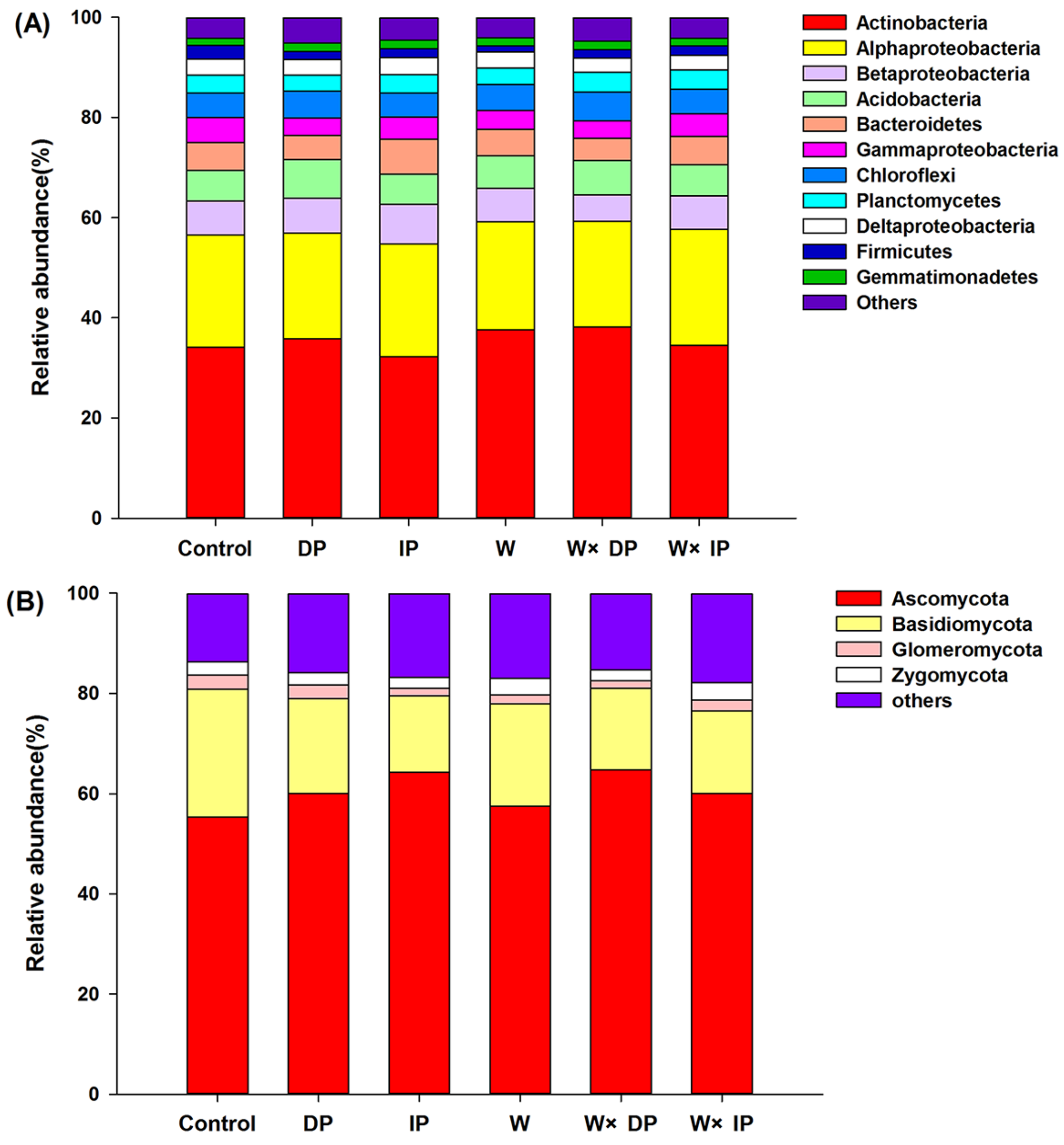

Figure S3: Response ratio method exhibited significant changes in abundance of bacteria OTUs at DP (A), IP (B), W (C), W×IP (D) relative to control.

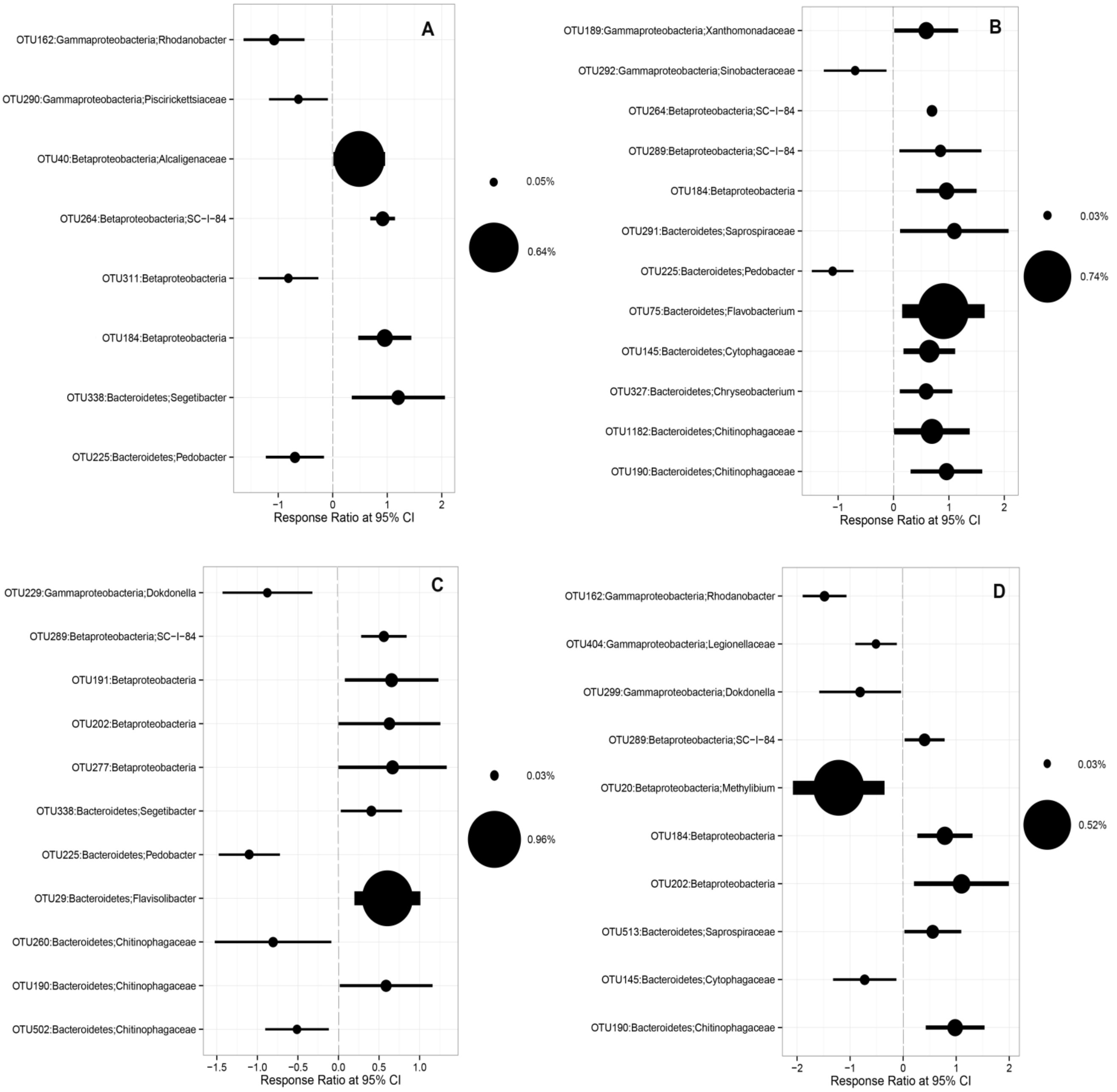

Figure S4: Effects of warming and altered precipitation treatments on plant community Shannon diversity (a) and Pielou evenness (calculated as J=H/log(S), H: Shannon index and S: total number of observed species in the community; b). Letters above the bars indicate statistical differences between control, warming and altered precipitation plots at Tukey's HSD for multiple comparisons ($p<0.05$, mean ±SE, n=3). DP: decreased precipitation for 50%; IP: increased precipitation for 50%; W: warming for 2 ℃; W×DP: warming for 2 ℃ and decreased precipitation for 50%; W×IP: warming for 2 ℃ and increased precipitation for 50%.

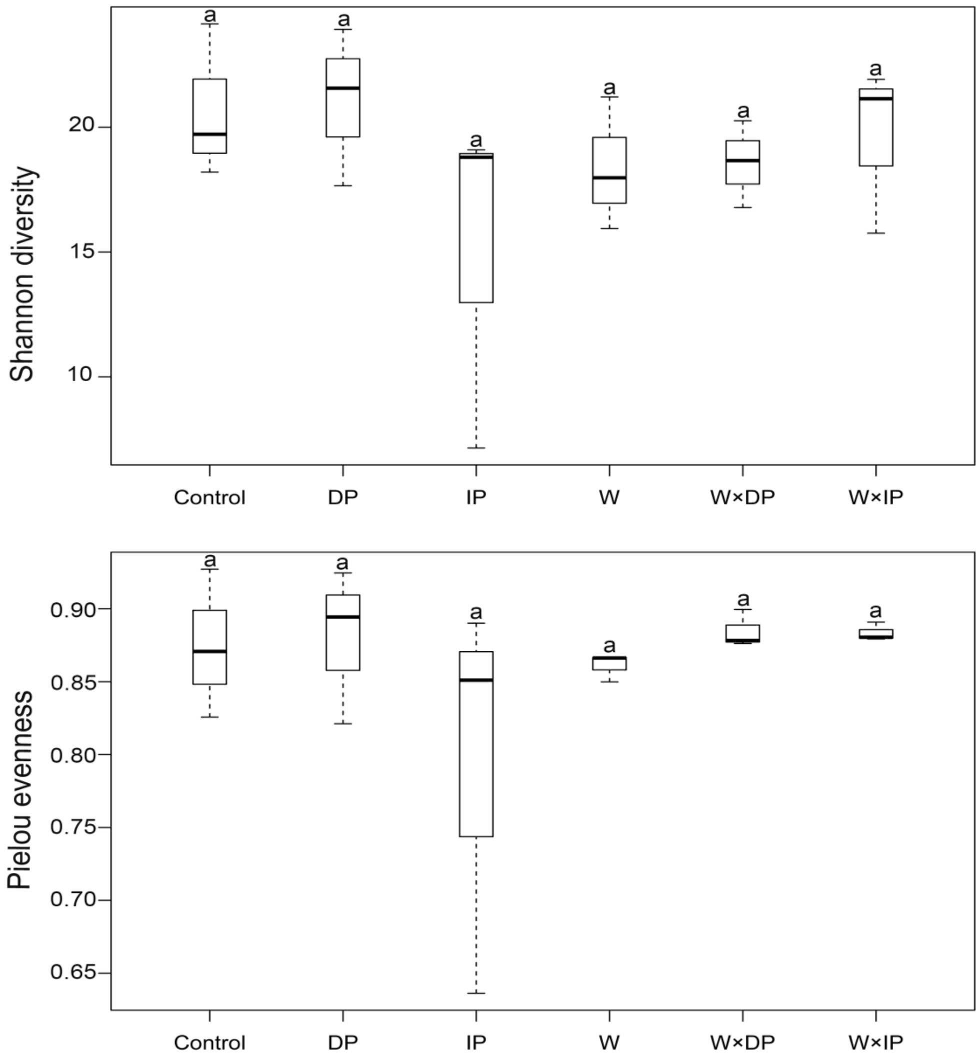

Supplement: Supplementary file 1 [file Presentation1.PDF]
